# Activated Clotting Time Level with Weight Based Heparin Dosing During Percutaneous Coronary Intervention and its Determinant Factors

**DOI:** 10.5681/jcvtr.2014.021

**Published:** 2014-06-30

**Authors:** Majid Soleimannejad, Naser Aslanabadi, Bahram Sohrabi, Morteza Shamshirgaran, Ahmad Separham, Babak kazemi, Ebrahim Khayati shal, Reza Madadi, Hamidreza Shirzadi, Hoda Azizi, Samad Ghaffari

**Affiliations:** Cardiovascular Research Center, Tabriz University of Medical Sciences, Tabriz, Iran

**Keywords:** Activated Clotting Time Heparin, Percutaneous Coronary Intervention

## Abstract

***Introduction:*** Percutaneous coronary intervention (PCI) may be associated with Thrombotic complications. Unfractionated heparin (UFH) is a potent and preferable antithrombotic agent during this procedure. Activated clotting time (ACT) is a good assay for accurate titration of UFH during PCI. The aim of this study was to evaluate ACT levels 10 minutes after administration of 100U/kg IV heparin and determining its associated factors.

***Methods:*** This study was performed in Madani hospital, Tabriz, Iran between January 2013 to January 2014. One hundred and two patients candidates for elective PCI were enrolled in the study. Data including demographic and risk factors were collected.

***Result:*** The range of ACT was between 165 to 750 seconds (mean 319.8 seconds), 52 (51%) patients had ACT levels lower than 300sec and 12 (11.8%) patients had ACT levels between 300 to 350 seconds which is known optimal range and 38 (37.2%) cases had ACT levels above this value. Major risk factors had no effect on ACT value, but there was a trend to higher levels with increasing age (P=0.06). There was no difference in the rate of major or minor bleeding with respect to ACT levels (P=0.52). There was a trend to higher rate of minimal bleeding in those with ACT >350 sec (P=0.06).

***Conclusion:*** Weight based UFH injection may result in suboptimal anticoagulation during the procedure. Routine ACT measurement may be necessary to ascertain adequate anticoagulation. Major risk factors had no effect on ACT level and it was not associated with the rate of bleeding.

## Introduction

Unfractionated heparin (UFH) is the primary antithrombotic agent for prevention of ischemic complications.^1^ Adequate dosing with UFH effectively suppresses the thrombin generation associated with balloon and stent induced intimal injury.^2^ Despite recent evolutions in anticoagulation therapy with improved outcome of PCI, considering its relative low cost, the availability of a rapid point of care for dosing (ACT) and a known antagonist that allows the prompt reversal of antithrombotic activity, UFH is yet an ideal option. In plasma heparin concentrations of between 0.1–1.0 U/ml, which is usual result of prolonged heparin infusion, aPTT is a sensitive and reliable test. However, in plasma heparin concentrations of more than 1.0 U/ml, the aPTT becomes prolonged beyond measurable levels. Therefore, the aPTT is not suitable for monitoring patients undergoing PCI where plasma levels may be in the range of 1.0–5.0 U/ml. For PCI, the activated clotting time (ACT) is the standard monitoring test.^3^ In the era of PCI with stent, periprocedural heparin dosing varies widely and optimal ACT level of 300-350 sec. with Hemochron device and 250-300 sec with Hemotec system is empiric.^4^ Our study, measures ACT with Hemochron device.

There are a lot of contributory factors which may affect the therapeutic impact of bolus administration of heparin. Clinical settings like acute coronary syndromes and presence of DM may lead to heparin resistance and body mass index may alter the volume of distribution of the drug and also concomitant use of some drugs like nitrates and thrombolytic agents may modify the response to a given dose of heparin.^5^ The narrow range of therapeutic effect of UFH in patients undergoing PCI mandates accurate ACT measurement.

Some trials used 10000 IU fixed dose UFH, irrespective of weight and BMI, and not only, the optimal ACT was seen in minority of patients but also most of ACT levels didn’t have the normal distribution.^6^ Other trials study the high dose 15000U UFH. The results were supraoptimal and bleeding complication was high. Few trials evaluate the relation between ACT levels and bleeding and ischemic complications.^6^ Surprisingly, there was weak correlation between ACT levels and bleeding and ischemia. Even in some of them, the bleeding complication was higher in low ACT levels and vice versa. Current PCI guidelines recommend low dose versus high dose heparin and per kg dosing vs. fixed dosing for PCI.^4^ Considering these controversial results and some other studies which revealed a race difference in ACT response for a given heparin dosage^7^, we designed current study. We used 100 U/kg heparin dosing to evaluate ischemic and hemorrhagic complications at the time of hospital stay**.**

## Materials and methods

The study was conducted in Shahid Madani heart center, affiliated to Tabriz University of medical sciences, Tabriz, Iran, between January 2013 to January 2014. Written informed consent was obtained from patients prior to the enrollment. In a cross- sectional descriptive analytic study we evaluated efficacy of fixed dose (100 U/kg) heparin dosing on elective PCI patients with ACT measurement using Hemochron device. We followed them for ischemic and hemorrhagic complications up to hospital discharge. One hundred and two patients were included and the blood samples for ACT were taken 10 minutes after heparin injection. Those admitted with ACS or receiving heparin for the last 8 hours or those with recent administration of GP IIb/IIIa inhibitors and candidates for primary PCI were excluded from the study. We defined severity of bleeding based on TIMI definitions. Minimal bleeding was defined as any clinically overt sign of hemorrhage that is associated with a fall in hemoglobin <3 g/dL, fall in hemoglobin between 3 to ≤5 g/dL was defined as minor bleeding and intracranial bleeding or those with a fall in hemoglobin of more than 5 g/dl was defined as major bleeding.^8^

## Results

A total of 102 patients were included in this study, the mean age of them was 58.4±9.4 years, with an age range of 33 to 77 years. The majority of them were male (66.7%) and about 80% were overweight or obese (Table 1).

**Table 1:** General characteristics of study population and common cardiovascular risk factors

		**No.**	**%**
**Age**	<50	15	14.9
	50-59	38	37.6
	≥60	48	47.5
**Gender**	Male	68	66.7
	Female	34	33.3
**Hyperlipidemia**		39	38.2
**Hypertension**		59	58.4
**Diabetes**		32	31.3
**Smoking**		19	18.6
**BMI**	Normal	19	18.8
	Overweight	58	57.4
	Obese	24	23.8
**Number of RF **	≤1	30	29.4
	≥2	72	70.6

BMI: Body Mass Index, RF: Risk Factor

In majority of cases (61 cases, 59.8%), PCI was done on LAD or its branches; in 11 (10.8%) on LCX or its branches; and in 30 patients (29.4%) on RCA; in more than two third of patients (85 cases, 83.3%) PCI was done on a single vessel; in 16 cases (15.7%) on two vessels; and in one case (1%) on 3 vessels.

Distribution of common cardiovascular risk factors varied by sex, 56% of women and 19% of men were diabetic (P=0.003), there was a trend to higher frequency of smoking among men [26.5% vs. 2.9%, (P=0.8)]. BMI of more than 30 was significantly more frequent in women [9 (13.2%) vs 15 (44.1%) P =0.001]. About seventy percent of our patients had at least two major coronary risk factors.

**Figure 1:**
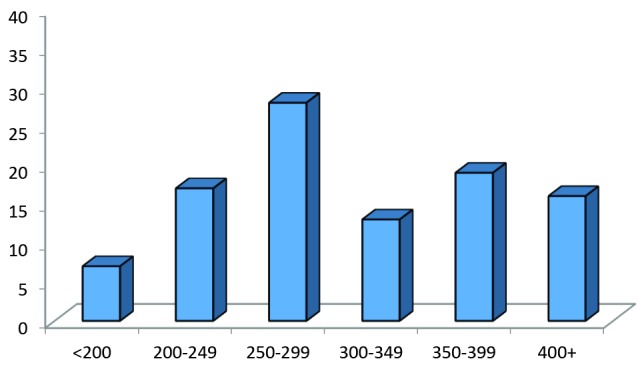
Distribution of ACT levels with weight based UFH administration

ACT range was between 165 to 750 seconds (mean 319.8±1.3.9 sec). Figure 1  shows the ACT levels, as it can be seen 52 (51%) of patients had ACT levels lower than 300 seconds which was considered suboptimal. Optimal level of ACT level (300-350 seconds) was observed in only 12 patients (11.8%) and 38 (37.2%) patients had supra-optimal level of ACT (> 350 sec).

Post procedure ischemic event was occurred in only one patient. The ACT value of this patient was above optimal value (450 sec). We had no TIMI major bleeding in our patients, minor bleeding was non-significantly higher in those with ACT value > 350 sec [2 (3.2%) vs 0, p=0.52], however, there was a trend to more minimal bleeding in those with ACT levels higher than 350 sec. [1 (1.6%) vs 4 (10.5%), P= 0.06].

We compared the means of ACT levels in different subgroups, ACT increased with increasing age from 285.4± 64.9 in <50 years of age to 346.3 ± 124.2 seconds in 60 years of age and above but this increase was not statistically significant. Mean of ACT was higher in male patients, in overweight/obese patients, in those who were hypertensive, diabetic, and patients with hyperlipidemia. However these differences were statistically non-significant (Table 2).

**Table 2:** Distribution of ACT levels by patients’ characteristics and risk factors

		**ACT**		P- value
		Mean	SD	
**Age** ** **	<50	285.4	64.9	0.06
	50-59	302.3	80.2	
	≥60	346.3	124.2	
**Sex** ** **	Male	323.1	91.7	NS
	Female	313.5	125.6	
**BMI** ** **	Normal	311.3	114.6	
	Overweight	316.1	83.8	NS
	Obese	336.4	139.9	
**Hypertension **	Yes	328.6	115.3	NS
	No	308.3	85.3	
**DM **	Yes	329.0	108.1	NS
	No	316.2	102.8	
**Hyperlipidemia **	Yes	341.4	124.2	NS
	No	306.0	86.9	
**Smoking **	Yes	307.6	87.3	NS
	No	322.5	107.5	
**Number of risk factors **	≤1	297.0	75.1	NS
	≥2	329.1	112.7	

BMI: Body Mass Index, DM: Diabetes Mellitus

## Discussion

The standard test for evaluation of the degree of anticoagulation activity is aPTT, but it needs laboratory equipment and trained staff, and it cannot be done as a bedside test.^6^ On the other hand, high dose of heparin used in PCI, results in aPTT values beyond the measurable range.^3^ ACT does not have these disadvantages, but there has been much controversy over the level of “optimal ACT for PCI. Early studies showed a linear relationship between heparin dose and ACT levels, but the slope of this line varies from patient to patient.^9^

Optimization of heparin dosing remains a major drawback for it and there has been much controversy over the level of optimal ACT for PCI. There is too much patient to patient variation between heparin dose and extent of ACT. So in most centers, it needs to administer added heparin to optimize ACT levels. On the other hand, there are some debates about a linear relationship between achieved ACT and ischemic or hemorrhagic endpoints. In the ESPIRIT trial, the incidence of ischemic events did not increase as ACT decreased, at least to a level of 200 s. The increase in major or minor bleeding corresponding with increasing ACT had not statistical significance in this trial.^10^ In PROLOG study, a strong trend toward a diminished risk of bleeding was shown with reductions in weight-adjusted heparin dosing and early sheath removal.^11^ Chew et al. in a meta analysis showed that the risk of ischemic end points was progressively reduced with increasing ACT levels, with no observed upper limit of efficacy. The ACT also correlated with bleeding events, with the lowest event rates seen in the range of 325 to 350 s.^1^ With an ACT level of 184 +/- 39 s and aggressive intravenous and oral platelet inhibition Capuano et al. reported acceptable results.^12^ Some of these controversial results may be related to antiplatelet regimens and especially IIb-IIIa inhibitors usage during intervention. Also improve in catheters and stents design and using vascular closure techniques may have a role in determining the risk of ischemic or bleeding complications.

In this study, ACT range was 165 To 750 seconds and almost half of the ACT levels were lower than 300 sec (suboptimal), and optimal value with such weight adjusted dosage was achieved only in 11.8% of patients.

In this small cohort of our patients there was no dramatic correlation between ACT measures and bleeding and ischemic complications except for a trend to higher minimal bleeding in those with higher ACT levels. There was a trend to higher ACT value in elderly patients but there was no significant difference between ACT levels in those with and without major coronary risk factors. Larger studies with higher sample sizes are needed to confirm this.

## Ethical issues

The study was approved by the Ethics Committee of the University.

## Competing interests

Authors declare no conflict of interest in this study.

## References

[1] Chew DP, Bhatt DL, Lincoff MA, Moliterno DJ, Brener SJ, Wolski KE (2001). Defining the optimal activated clotting time during percutaneous coronary intervention: aggregate results from 6 randomized, controlled trials. Circulation.

[2] Narins CR, Hillegass WB, Nelson CL (1996). Relation between activated clotting time during angioplasty and abrupt closure. Circulation.

[3] Marmur JD (2002 Apr). Direct versus indirect thrombin inhibition in percutaneous coronary intervention. J Invasive Cardiol.

[4] Lee MS, Rametta A, Aragon J, Khan A, Wilentz J, Singh V (2005). New heparin dosing regimen for diabetes undergoing percutaneous coronary intervention. J Invasive Cardiol.

[5] Niccoli G, Banning AP (2002). Heparin dose during percutaneous coronary intervention: how low dare we go?. Heart.

[6] Maadani M, Abdi S, Zahedmehr A (2008). Assessment of safety and efficacy of conventional heparin dose in percutaneous coronary interventions characterized by means of activated clotting time. Iranian Heart Journal.

[7] Shimada YJ, Nakra NC, Fox JT, Kanei Y (2010). Relation of race (Asian, African-American, European-American, and Hispanic) to activated clotting time after weight-adjusted bolus of heparin during percutaneous coronary intervention. Am J Cardiol.

[8] Mehran R, Rao SV, Bhatt DL, Gibson CM, Caixeta A, Eikelboom Eikelboom (2011). Standardized bleeding definitions for cardiovascular clinical trials: a consensus report from the Bleeding Academic Research Consortium. Circulation.

[9] Mulry CC, Leveen RF, Sobel M, Lampe PJ, Burke DR (1991). Assessment of heparin anticoagulation during peripheral angioplasty. J Vasc Interv Radiol.

[10] Tolleson TR, O’Shea JC, Bittl JA, Hillegass WB, Williams KA, Levine G (2003). Relationship between heparin anticoagulation and clinical outcomes in coronary stent intervention: observations from the ESPRIT trial. J Am Coll Cardiol.

[11] Lincoff AM, Tcheng JE, Califf RM, Bass T, Popma JJ, Teirstein PS (1997). Standard versus low-dose weight-adjusted heparin in patients treated with the platelet glycoprotein IIb/IIIa receptor antibody fragment abciximab (c7E3 Fab) during percutaneous coronary revascularizationPROLOG Investigators. Am J Cardiol.

[12] Capuano C, Sesana M, Leonzi O, Cuccia C (2006). Safety and efficacy of low-dose unfractionated heparin during percutaneous coronary revascularisation and the relationship between activated clotting time and haemorrhagic or ischaemic complications: our results. J Cardiovasc Med (Hagerstown).

